# Determinants of adolescent pregnancy in sub-Saharan Africa: a systematic review

**DOI:** 10.1186/s12978-018-0460-4

**Published:** 2018-01-27

**Authors:** Ibrahim Yakubu, Waliu Jawula Salisu

**Affiliations:** 1 School of Public Health, Tehran University of Medical Sciences, International Campus, Tehran, Iran; 2 School of Nursing and Midwifery, Tehran University of Medical Sciences, International Campus, Tehran, Iran

**Keywords:** Adolescent pregnancy, Determinants, Sub-Saharan Africa

## Abstract

**Background:**

Adolescent pregnancy has been persistently high in sub-Saharan Africa. The objective of this review is to identify factors influencing adolescent pregnancies in sub-Saharan Africa in order to design appropriate intervention program.

**Methods:**

A search in MEDLINE, Scopus, Web of science, and Google Scholar databases with the following keywords: determinants, factors, reasons, sociocultural factors, adolescent pregnancy, unintended pregnancies, and sub- Saharan Africa. Qualitative and cross-sectional studies intended to assess factors influencing adolescent pregnancies as the primary outcome variable in sub- Saharan Africa were included. Our search was limited to, articles published from the year 2000 to 2017 in English. Twenty-four (24) original articles met the inclusion criteria.

**Results:**

The study identified **Sociocultural, environmental and Economic factors** (Peer influence, unwanted sexual advances from adult males, coercive sexual relations, unequal gender power relations, poverty, religion, early marriage, lack of parental counseling and guidance, parental neglect, absence of affordable or free education, lack of comprehensive sexuality education, non-use of contraceptives, male’s responsibility to buy condoms, early sexual debut and inappropriate forms of recreation). **Individual factors** (excessive use of alcohol, substance abuse, educational status, low self-esteem, and inability to resist sexual temptation, curiosity, and cell phone usage). **Health service-related factors** (cost of contraceptives, Inadequate and unskilled health workers, long waiting time and lack of privacy at clinics, lack of comprehensive sexuality education, misconceptions about contraceptives, and non-friendly adolescent reproductive services,) as influencing adolescent pregnancies in Sub-Saharan Africa

**Conclusion:**

High levels of adolescent pregnancies in Sub-Saharan Africa is attributable to multiple factors. Our study, however, categorized these factors into three major themes; sociocultural and economic, individual, and health service related factors as influencing adolescent pregnancies. Community sensitization, comprehensive sexuality education and ensuring girls enroll and stay in schools could reduce adolescent pregnancy rates. Also, provision of adolescent-friendly health services in schools and healthcare centers and initiating adolescent empowerment programs could have a positive impact.

## Plain English summary

Adolescent pregnancies have been persistently high in sub-Saharan Africa. This study seeks to identify factors influencing adolescent pregnancies in sub-Saharan Africa through a systematic review of published scientific articles.

A total of two hundred and twenty nine (229) original articles published between 2000 and 2017 were first identified from various data bases. Finally, twenty-four (24) original articles met the inclusion criteria and were included in the study. All articles were studies conducted in sub-Saharan Africa.

The study identified **Sociocultural, environmental and Economic factors** (Peer influence, unwanted sexual advances from adult males, coercive sexual relations, unequal gender power relations, poverty, religion, early marriage, lack of parental counseling and guidance, parental neglect, absence of affordable or free education, lack of comprehensive sexuality education, non-use of contraceptives, male’s responsibility to buy condoms, early sexual debut and inappropriate forms of recreation). **Individual factors** (excessive use of alcohol, substance abuse, educational status, low self-esteem, and inability to resist sexual temptation, curiosity, and cell phone usage). **Health service-related factors** (cost of contraceptives, Inadequate and unskilled health workers, long waiting time and lack of privacy at clinics, lack of comprehensive sexuality education, misconceptions about contraceptives, and non-friendly adolescent reproductive services,) as influencing adolescent pregnancies in Sub-Saharan Africa.

We believe that Community sensitization, sex education and ensuring girls enrol and stay in schools could reduce adolescent pregnancy rates. Also, provision of adolescent friendly health services at schools and initiating adolescent empowerment programs could have positive impact.

## Background

The long-lived belief in the African society where females were not prioritized for education is fading out. With this, it expected that female education will increase in sub-Saharan Africa [1]. Unfortunately, adolescent pregnancy contributes to denying brilliant students education and has potential to retard their growth and development including that of their children.

According to WHO about 17 million adolescent girls give birth every year and most of these births occur in low- and middle-income countries [2]. Adolescent health and development are of global concern. The need to prevent early pregnancy among adolescent girls in Sub-Saharan Africa has been recognized increasingly over recent years [3]. African countries lead the world in teen pregnancies: With Niger on the top list of 203.604 births per 100,000 teenage women. Mali follows with 175.4438, Angola (166.6028), Mozambique (142.5334), Guinea (141.6722), Chad (137.173), Malawi (136.972), and Cote d’Ivoire (135.464) [4].

Adolescent girls continue to experience the disproportionately high burden of sexual and reproductive ill health, particularly in Sub-Saharan Africa [3]. High adolescent pregnancies with adverse health and social consequences are urgent problems facing low- and middle-income countries [2]. Adolescents are likely to have complications of pregnancy including unsafe abortion and more likely to become young mothers a second time [2, 5, 6]. Their infants are also more likely to be born premature and to die in the perinatal period [7]. Babies born to adolescent mothers face a substantially higher risk of dying than those born to women aged 20 to 24 [2, 5, 8]. They are at risk of malnutrition, low mental and physical development, inappropriate social connection with parents and poor education [5, 9].

Adolescents develop psychological problems from social stigma, suffer physical and domestic violence in their attempt to meet the demands of pregnancy and childbearing [9, 10]. Also, they most likely would drop out and may not get the chance to return to school [11]. The inadequate resources of low and middle-income countries would have to be channeled to cater for the health needs of pregnant and teen mothers including their children [5]. Economic opportunities are limited to adolescents who could not complete school because of unintended pregnancies. This could be the beginning of a poverty cycle in families, however, some are able to face the challenge and become productive later in life.

Factors associated with unintended pregnancies amongst adolescents are early marriages, culture, religion, gender [12], poor social and economic support [13, 14]. Curiosity and peer pressure [15, 16], lack of comprehensive sexuality education [17–19], poor reproductive health services provision [19, 20], poor attitude of health workers to providing contraceptive services for adolescents [15, 21]. Also, unmet need for contraceptives by adolescents [22] and fear of contraceptive side effects [16]. Barriers to contraceptive use among adolescents include inadequate sexual knowledge and risk perceptions. Also, lack of skills and power to negotiate safer sex options, ambivalence towards sex, and negative social norms around premarital sexual activity and pregnancy [23].

Policy makers in Sub-Saharan Africa need to understand the determinants of adolescent pregnancy in their context in order to design pragmatic interventional programs to reduce unintended pregnancies amongst adolescents. Since there has not been any review of literature on the determinants of adolescent pregnancy in Sub-Saharan Africa, this study aims to identify the determinants of high adolescent pregnancy in Sub-Saharan Africa.

## Methods

### Search strategy

MEDLINE, Scopus, web of science and Google Scholar databases were searched in July 2017 with the following keywords: determinants, factors, reasons, sociocultural factors, adolescent pregnancy, unintended pregnancies, and sub- Saharan Africa. Qualitative and cross-sectional studies intended to assess the factors influencing adolescent pregnancies either intended or unintended as the primary outcome variable in sub- Saharan Africa was included. Our search was limited to articles published in English from 2000 to 2017.

### Inclusion and exclusion criteria

Qualitative and cross-sectional studies that assessed the factors associated with adolescent pregnancy, conducted in sub-Saharan Africa, whether the pregnancy was intended or not, from the year 2000 to 2017 were included. Studies that addressed factors associated with adolescent pregnancy, yet conducted outside Sub-Saharan Africa were excluded from the study.

### Identification of reviews

**Fig. 1:**
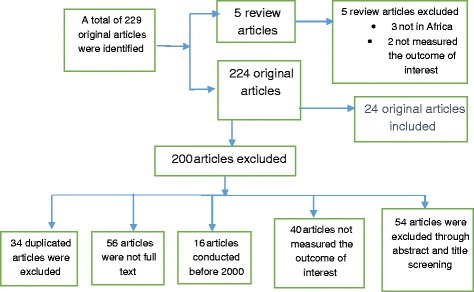
Inclusion and exclusion criteria

A search framework was constructed and implemented through a broad scope and exhaustive search using Tehran University of Medical Sciences electronic library to identify applicable studies published in English. The search identified a total of 229 articles, which composed of 244 original research articles and 5 review articles. After the screening of titles and abstracts, 54 articles were excluded because they did not address adolescent pregnancy. Articles that addressed adolescent pregnancy were 170 and 5 reviews. All the review articles were excluded because 3 were conducted outside the study setting, and the other 2 did not assess the determinants of adolescent pregnancy. With the rest of the articles, 40 did not assess determinants of adolescent pregnancy, 16 were conducted before the year 2000, 56 were not full text and 34 were duplicated. Therefore, 24 research articles were sieved and included in the study. Figure 1 demonstrates articles selection criterion.

### Variables assessed

The main variables assessed were age, lack of money, lack of family support, the culture of not talking about sex at home, peer influence, broken homes, early marriages, and religion. Service-related factors such as lack of adolescent-friendly services, inadequate comprehensive sexuality education, non-availability and cost of contraceptives, inadequate health personnel, judgmental attitude of service providers and inadequate counseling. Personal behavioral factors such as alcohol and tobacco use, fear of stigma and being judged by the service provider, low self-efficacy, low self-esteem, vulnerability and rape, curiosity, inadequate education and knowledge about contraceptives.

### Articles appraisal

The Joanna Briggs appraisal tool [24] was used independently by the authors to appraise and certify for inclusion or exclusion of articles. Both authors, before inclusion, reached consensus. The tool consists of a checklist of ten questions for qualitative studies, eight for cross-sectional studies and 11 for systematic reviews and research syntheses.

### Strengths of the study

The study has revealed, through a comprehensive search the determinants of adolescent pregnancy in Sub-Saharan Africa. The results of the study are similar to global reports [25]. In addition, the study revealed a gap in research regarding literature on determinants of adolescent pregnancy in the five sub-Saharan Africa countries with the highest rates [4].

### Weaknesses of the study

Diverse disciplines deal with adolescents and adolescent pregnancies; this makes it possible to miss some articles during the search process since their findings may not be published in scientific based journals which were our main source of data. Secondly, grey literature, reports, and unpublished studies were not included in this review. In addition, some articles reported findings from small sample sizes.

### Operational definition of concepts

#### Adolescent reproductive services

Healthcare services offered to assist adolescents access sexual and reproductive health information.

#### Comprehensive sexuality education

Teaching abstinence as the best method for avoiding unintended pregnancy, but also teaching about condoms and contraception to reduce the risk of unintended pregnancy. It also involves empowering adolescents to resist sexual temptations and peer pressure.

#### Curiosity

A strong desire to discover new things, especially about sex and relationships.

#### Early marriage

Union between two people in which one or both parties are younger than 18 years of age:

#### Early sexual debut

Having had first sexual intercourse at or before age 14.

#### Excessive use of alcohol

Uncontrolled and widespread alcohol usage.

#### Family dysfunction

Unhealthy interactions, conflict, misbehaviour, and child neglect of parents.

#### Gender power relations

The culturally determined social status of men and women in relationships.

#### Inability to resist sexual temptation

Inability to avoid or say no to sexual desires and pressure from both internal and external sources, and acting on it.

#### Inappropriate recreation

Recreational activities, which creates a risky sexual environment e.g. disco dances, clubbing etc.

#### Low self-esteem

Lacking self-confidence to turn down sexual advances from men.

#### Peer influence

Social pressure by members of one’s peer group to take a certain action, adopt certain values, or otherwise conform in order to be accepted.

#### Positive attitude towards early sexual relationships

Having a good feeling or emotion towards early sexual relationships.

#### Religion

A system of faith and worship.

#### Sexual advances

Purposeful visual, verbal or physical conduct of a sexual nature.

#### Substance abuse

Excessive and uncontrolled use of illicit and addictive substances such as tobacco, marijuana**.**

## Results

Articles included in this review were studies conducted in Sub-Saharan African countries with a focus on adolescent pregnancies.

Out of 24 articles, eight (8) were qualitative research [19, 23, 26–31], 15 were cross-sectional studies [15, 32–45], and one article used mixed method [46] (Table 1).

**Table 1:** Main determinants of unintended pregnancy amongst adolescents

Title	Year/country	Study type	Determinants of adolescent pregnancy
Understanding sexual and reproductive health needs of adolescents: evidence from a formative evaluation in Wakiso district, Uganda	2015 Uganda	Qualitative study	Sexual advances from adult males, rape, defilement, alcohol, marijuana, lack of youth counsellors, lack of sex education, inadequate education on family planning, cost of contraceptives, inadequate health workers and fear of stigma in seeking help form health workers.
Barriers to access reproductive healthcare for pregnant adolescent girls: a qualitative study in Tanzania	2015 Tanzania	Qualitative study	They viewed condoms as ineffective for preventing STIs and pregnancies and unnecessary for those in committed relationships. Stigma and long waiting times. Lack of privacy in the clinics discouraged young females from seeking reproductive healthcare, lack of privacy, unkind health care workers
Relationship dynamics and teenage pregnancy in South Africa	2001 south Africa	Exploratory study	Forced sexual initiation, unwillingness to confront unfaithful partner, partners of adolescents were older, unequal power relations, living in extended family, non-use of contraceptives, peer influence, and curiosity.
Who’s that girl? A qualitative analysis of adolescent girls’ views on factors associated with teenage pregnancies in Bolgatanga, Ghana	2016 Ghana.	Qualitative study	Material gain, positive attitude towards relationship, early sexual debut, peer influence, most parents don’t talk about safe sex with their children, male should be responsible to buy condoms, fear of ridicule, misconceptions about family planning, inability to resist temptation,
Gendered norms, sexual exploitation and adolescent pregnancy in rural Tanzania	2013, Tanzania	Qualitative study	Poverty, inability to exercise control over sexual decisions, adolescents sexual relationship with older men, early marriage, sexual expectations from men, rape, coerced sex
Adolescent pregnancy and associated factors in South African youth	2012 south Africa	Cross sectional study	Unemployment, poverty, high sexual permissive attitude, contraceptive use, didn’t understand the risk of pregnancy, to prove their maturity, unequal power in relationship
Family and community support to adolescent mothers in Swaziland	2003 Swaziland	Mixed method	Peer influence, lack of sexual and reproductive information from families and communities
Factors influencing the adolescent pregnancy rate in the Greater Giyani Municipality, Limpopo Province – South Africa	2015 south Africa	Descriptive and explorative survey	Inconvenient health services, poor relationship with health workers, peer pressure, inadequate sexual knowledge, changing attitude toward sex.
Determinants of Sexual Activity and Pregnancy among Unmarried Young Women in Urban Kenya: A Cross-Sectional Study	2015 Kenya	Cross-Sectional Study	Education level, religion, employment status, household size, family planning knowledge, misconceptions, and early sexual debut.
Unmet social needs and teenage pregnancy in Ogbomosho, South-western Nigeria	2014 Nigeria	Cross-Sectional Study	The unmet material and financial supports expected from parents, the lack of free education from government up until secondary school level, the lack of sex education and knowledge needs for signs of maturity, and discouragement from friends not to have boyfriend.
Adolescent girls, illegal abortions and “sugar-daddies” in Dar es Salaam: vulnerable victims and active social agents	2001 Tanzania	Qualitative study	Material benefits from men, high-risk sexual activity, lack of family planning information, sex education and poor health services.
Teenage pregnancy experiences in rural Kenya	2003 Kenya	Community based survey	Sexual active adolescents, unmet reproductive health needs, education level of adolescents
Health workers’ attitudes toward sexual and reproductive health services for unmarried adolescents in Ethiopia	2012 Ethiopia	descriptive cross-sectional survey	Negative attitude of health workers towards providing RH services to unmarried adolescents, low education of health workers, and lack of training on RH services.
Nurse-Midwives’ Attitudes towards Adolescent Sexual and Reproductive Health Needs in Kenya and Zambia	2006 Kenyan and Zambia	cross-sectional survey	Negative attitudes of health workers towards adolescent sexual activity, contraceptive use and seeking reproductive health services.
Determinants of teenage pregnancies: The case of Busia District in Kenya	2007 Kenya	Cross sectional survey	Level of education, sex education, peer pressure, inappropriate form of recreation, lack of parental guidance and counselling, poverty, knowledge of contraception.
Blood Blockages and Scolding Nurses: Barriers to Adolescent Contraceptive Use in South Africa	2006 south Africa	Qualitative study	Pressure from male partners, fears about the effects of contraception, health workers attempt to stigmatize teenage sexuality, scolding and hash treatment of adolescents, unwillingness to acknowledge adolescent experience as contraceptive users.
A participatory action research approach to developing youth-friendly strategies for the prevention of teenage pregnancy	2016 South Africa	participatory action research approach	Sexual curiosity, alcohol consumption, unprotected sex, peer influence, lack of family support, parental absence, low family socioeconomic status, gender power inequality, relationship with elder men, lack of youth-friendly clinics
Socio-Cultural Determinants of Contraceptives Use Among Adolescents in Northern Ghana	2015 Ghana	Descriptive cross-sectional study	Early sexual debut, educational level, parental neglect, money, curiosity, peer pressure
Predictors of Sexual Debut Among Young Adolescents in Nairobi’s Informal Settlements	2014 kenya	Descriptive cross-sectional study	School dropout, education, severe family dysfunction, lack of parental control.
Early Pregnancy of Junior High School Girls: Causes and Implications on Academic Progression in the Talensi District of the Upper East Region Of Ghana	2015 Ghana	Cross-sectional study	Cell phone usage by teenagers, inadequate contraceptives, peer group influence, family neglect and poverty, peer group influence, lack of sex education.
Teenage Pregnancy in the Builsa District: A Focus Study in Fumbisi.	2013 Ghana	Cross-sectional study	Poverty, prostitution, inadequate sex education, inadequate family support.
The cause and effect of teenage pregnancy: case of kontagora local government area in niger state, northern part of nigeria	2013 Nigeria.	Cross-sectional study	Early sexual debut, socio-economic background, early marriage and traditional gender roles, peer pressure, lack of sex education and non-used of contraceptive during sexual intercourse.
Poverty the Cause of Teenage Pregnancy in Thulamela Municipality	2015 south Africa	Cross-sectional study	Poverty, low socio-economic family status, lack of parental support, inadequate sex education from parents, peer influence.
The Effects of Teenage Pregnancy on the Educational Attainment of Girls at Chorkor, a Suburb of Accra	2013 Ghana	Qualitative study	Poor parenting, poverty, peer influence and school dropout.

Participants in this study were mostly adolescents. The study settings were both rural and urban, with an approximated total population of 11,651 participants. Participants per study varied from 10 as the least to 3122 as the highest. Refer to Table 2 for a detailed description of participant’s characteristics.

**Table 2:** Participants Characteristics

Author	Study setting/country	Number of participants	Gender % (n)	Age range (years)	Ethnicity	Socio-economic status	Marital status
Atuyambe et al. (2015) [19]	Wakiso district/Uganda	156	Females 50.6% (79), Males 49.4% (77)	10--19	not described	No employment status described	not indicated
Hokororo et al. (2015) [27]	Mwanza region/Tanzania	49	Females 100% (49)	15-20	Sukuma tribe	Non was employed	Legally married (2) Living with partner/boyfriend (40) Single or not living with partners (7)
R. Jewkes et al. (2001) [35]	Gugulethu and Khayelitsha, Cape Town/South Africa	544	Females 100% (544)	below 19 years	not described	No employment status described	not indicated
Krugu et al. (2016) [23]	Bolgatanga Municipality/Ghana	20	Females 100% (20)	14-19	not described	High school students	non was married
McCleary-Sills et al. (2013) [28]	Tanzania	82	Females 100% (82)	12--17	not described	No employment status described	not indicated
G. Mchunu et al. (2012) [38]	Eastern Cape, Gauteng, KwaZulu-Natal and Mpumalanga/ South Africa	3123	Female 45.4% (1418), Males 54.6% (1705)	18-24	not described	No employment status described	not indicated
P.T. Mngadi et al. (2003) [46]	Mbabane/Swaziland	31	Females 100% (31)	15-19	not described	Non was employed	not indicated
Mushwana et al. (2015) [15]	Greater Giyani Municipality/South Africa	147	Females 100% (147)	10--19	not described	No employment status described	Married (5), Single (136), Other (4)
Okigbo & Speizer (2015) [40]	Nairobi,Mombasa,Kisumu,Machakos, Kakamega/Kenya	2020	Female 100% (2020)	15-24	not described	Not employed 467, Student 658, Employed 895	non has ever been married
Salami et al. (2014) [41]	Ogbomosho, Oyo State/Nigeria	participants 174, key informants 12	Females 100% (174 + 12)	10-20 and above	not described	Not employed 34, Student 62, Trading 45, Others 33	not indicated
Silberschmidt & Rasch (2001) [29]	Dar es Salaam/Tanzania	51	Females 100% (51)	15-19	Not specific	Students 25, Employed 26	non was married
Taffa et al. (2003) [42]	Nyanza region/Kenya	1247	Females 100% (1247)	12--19	Not specific	students 233, not in school 331	married or co-habiting (253), not married (331)
Tilahun et al. (2012) [43]	Eastern Hararghe, Oromia region/Ethiopia	394	Females 301 (301), Males 23.6% (93)	18-24 (219), 25-35 (143), 36 and above [32]	Oromiffa	Nurses (119), Health Extension Workers (236), Health Assistants [21]	Married (245), Single (149)
Warenius et al. (2006) [44]	Kenya and Zambia	707	Females 92% (651), Male 8% (56)	22-60	Not specific	Enrolled Nurses (502), Registred Nurses (200), Dispensary Techs [5]	not indicated
M. Were (2007) [45]	Busia District/Kenya	258	Females 78.7% (203), Male 21.3% (55)	10--19	not described	No employment status described	not indicated
K Wood & R Jewkes (2006) [30]	Limpopo Province/South Africa	35	Females 100% (35)	14-20	Not specific	No employment status described	non was married
L. Wooda & F. Hendricks (2016) [31]	South Africa	24	Females 58.3% (14) Males 41.7% (10)	Below 18 years	not described	grade 11 learners	not indicated
A. Yidana et al. (2015) [32]	Yendi Municipality /Ghana	400	Females 62.8% (251), Males 37.3% (149)	14-19	Dagomba	No employment status described	Cohabiting (19), Divorced (1), Married (76), Single (292), Widowed (12)
M. Marston et al. (2013) [37]	Korogocho and Viwandani/Kenya	1754		12--16	Swahili	No employment status described	not indicated
E. Alhassan (2015) [34]	Talensi District/Ghana	310	Females 100% (310)	not specific	Telensi	Junior High School Students	not indicated
S. P. Adzitey et al. (2013) [33]	Fumbisi, Builsa District/Ghana	20	Females 100% (20)	14-20	Builsa (65%), Kasena (15%), Mamprusi (10%), Bimoba (5%) and Sisala (5%)	Students (95%), No education (5%)	Married (19), Single (1)
Ogori et al. (2013) [39]	Kontagora LocalGovernment Area, Niger State/Nigeria	40	Not specific	Not specific	Not specific	No employment status described	not indicated
Lambani M.N (2015) [36]	Limpopo Province/South Africa	10	Females 10% (10)	17-18	not described	Non was employed	not indicated
Gyan C. (2013) [26]	Chorkor, Greater Accra Region/Ghana	55	Females 100% (55)	not specific	Ga-Dangme and Akan	No employment status described	not indicated

At least 12 key informants composed of parents, school teachers, health providers, and adolescent mothers/fathers were involved in some of the included studies. They provided information regarding some of the determinants of adolescent pregnancy.

It is interesting to note that there is no published data within this review years from the top five sub-Saharan African countries with adolescent pregnancy above 140 births per 100,000 adolescents [47].

The study revealed three major themes influencing adolescent pregnancy in sub-Saharan Africa: *Sociocultural, environmental and Economic, Individual, and Health-Related Factors.*

### Sociocultural, environmental and economic factors

Peer influence was reported by 11 studies [15, 23, 26, 31, 34–36, 39, 41, 45, 46], Unwanted sexual advances from adult males which often led to coercive sexual relations [19, 28, 31, 35]. Also, unequal gender power relations [19, 27, 28, 30, 31, 35, 38, 39], poverty [19, 23, 26, 28, 31–34, 36, 38, 41, 45], religion and early marriage [28, 39, 40]. In addition, lack of parental counseling and guidance, severe family dysfunction with parental neglect [23, 26, 31, 33–37, 40, 41, 45, 46]. The absence of affordable or free education [41]. Lack of comprehensive sexuality education, both in schools and at home with family members [15, 19, 23, 26, 29, 31, 32, 34, 36, 37, 41, 45, 46]. Lack of knowledge, misconceptions, and non-use of contraceptives [19, 23, 29, 30, 35, 38, 40, 44, 45], male’s responsibility to buy condoms [23]. Positive attitude towards early sexual relationships, and early sexual debut [14, 22, 29, 31, 37–39, 41]. Inappropriate forms of recreation [45].

### Individual factors

Excessive use of alcohol and substance abuse [19, 31], educational status [26, 32, 37, 42], low self-esteem and inability to resist sexual temptation [23, 28, 30, 31, 35, 38, 39], and curiosity [31, 32, 35]. Cell phone usage by teenagers [34].

### Health service-related factors

Cost of contraceptives [19]. Inadequate and unskilled health workers [19, 27, 43]. Long waiting time and lack of privacy at clinics [27], lack of comprehensive sexuality education, and misconceptions about contraceptives [15, 19, 23, 27, 29, 30, 34, 35, 38, 40, 45, 46]. Also, non-friendly adolescent reproductive services, and negative attitude of health workers towards providing reproductive health services for adolescents [15, 19, 27, 29, 43, 44].

## Discussion

Sociocultural, economic, individual and health service factors were identified as the main determinants of adolescent pregnancy. These factors were found to influence high rates of adolescent pregnancy in sub-Saharan Africa, similar to the developed world [25].

A study by Fearon et al. reported peers to be influential in romantic and sexual behaviors of adolescents [48]. Their finding is consistent with the findings of this review. Studies from Ghana, Nigeria, Swaziland, Kenya, Tanzania, and South Africa reported the influence of peers in adolescent pregnancy. Particularly mentioned in a study from Nigeria [41], peers encourage their friends to get boyfriends.

Low socioeconomic status of parents makes adolescents vulnerable to unintended pregnancies since the means to afford basic needs, and sometimes contraceptives is a challenge. Some adults take advantage of this situation to provide basic needs to unsuspecting adolescents and engage in sexual relationships with them. This creates a power difference between adolescents and their adult partners making them powerless to negotiate for safer sex. The effect of this is teenage pregnancy and the spread of sexually transmitted infections. Studies from Ghana [23], South Africa [31] and Tanzania [29] demonstrate how poverty leads adolescents to engage in sexual relations with elderly men in order to meet their basic needs. Lambani [36], reported that adolescents intentionally get pregnant to receive government support intended for teenage mothers to improve their economic condition not considering the consequence of their action.

Lack of parental counseling and guidance, severe family dysfunction with parental neglect were found as risk factors for adolescent pregnancies [26, 33, 35, 40, 45]. Parental counseling and guidance improves communication between parents and adolescents and enables parents to address challenges of adolescents. Improved family communication and parent involvement in adolescents pregnancy prevention programs could delay adolescent sexual activity and pregnancy [49].

Inadequate knowledge, misconceptions and non-use of contraceptives [15, 19, 23, 26, 29, 31, 32, 34, 36, 37, 41, 45, 46] were identified as determinants of adolescent pregnancy. As indicated by Wood and Hendricks [31], health practitioners don’t relate health education to sociocultural context of adolescents but rather on bio-medical facts and warn of negative consequences. They do not as well explore their fears regarding contraception; therefore, adolescents do not feel the impact of comprehensive sexuality education. Uninformed adolescents perceive contraceptives as a reserve for married couples [29].

Inappropriate modes of recreation in the form of unmonitored nightclubs or mixed-sex partying. These expose adolescents to early sex since they socialize easily with men [45].

We found the positive attitude towards early sexual relationship and early sexual debut as factors contributing to adolescent pregnancy, consistent with findings from some developed countries [50–52]. The participants mostly lived in a social environment where adolescents had free will to choose sexual partners at an early age without much criticism from parents, caregivers, and peers [23]. In other reports, adolescents intentionally became pregnant as proof of love and commitment to their sexual partners [30].

Religion and early marriages were also found to contribute to high reports of adolescent pregnancies, which is consistent with a study by Parsons et al. [53]. Adolescents affected by early marriages are deprived of economic empowerment and self-efficacy and are at risk of early pregnancies [3]. They are also prone to maternal morbidity and mortality [54]. WHO’s guidelines on prevention of unintended pregnancy stressed on policies to reduce early marriage [6]. Rape, coerced sex, sexual advances from adult men and unequal gender power in relationships identified in this review, undermines adolescents’ decision-making ability to either reject sex or negotiate the use of contraceptives [55].

Individual factors that influence adolescent pregnancies include excessive use of alcohol and substance abuse. This behavior makes adolescent girls vulnerable, and an easy target for sexual exploits. This is consistent with previous findings which reported an association between high-risk sexual behavior, adolescent pregnancy and substance abuse [56]. Cell phone usage promotes easy communication among peers and their partners and also gives them easy access to the internet which they use without regulation, to surf explicit content motivating early sex [34].

Health service-related factors include the cost of contraceptives [19], healthcare centers lacking the adequate and skilled staff to attend to adolescents who need reproductive health services [19, 27, 43]. Long waiting time and lack of privacy at clinics discourage adolescents from visiting the facilities for services [27]. Also, inadequate comprehensive sexuality education and misconceptions about contraceptives [15, 19, 23, 27, 29, 30, 34, 35, 38, 40, 45, 46] were identified. Similarly, lack of friendly adolescent reproductive services and negative attitude of health workers towards providing reproductive health services for adolescents [15, 19, 27, 29, 43, 44] were all associated with adolescent pregnancy.

## Conclusion

High levels of adolescent pregnancies in Sub-Saharan Africa is attributable to multiple factors. Our study, however, categorized these factors into three major themes; **Sociocultural, environmental and Economic factors** (Peer influence, unwanted sexual advances from adult males, coercive sexual relations, unequal gender power relations, poverty, religion, early marriage. In addition, lack of parental counseling and guidance, parental neglect, the absence of affordable or free education, lack of comprehensive sexuality education, misconceptions, and non-use of contraceptives, male’s responsibility to buy condoms, positive attitude towards early sexual relationships, early sexual debut and inappropriate forms of recreation). **Individual factors** (excessive use of alcohol, substance abuse, educational status, low self-esteem, and inability to resist sexual temptation, curiosity, and cell phone usage). **Health service-related factors** (cost of contraceptives, Inadequate and unskilled health workers, long waiting time and lack of privacy at clinics, lack of comprehensive sexuality education, misconceptions about contraceptives, non-friendly adolescent reproductive services, and negative attitude of health workers towards providing reproductive health services for adolescents) as influencing adolescent pregnancies.

Seemingly unique to sub-Saharan Africa, our study found determinants of adolescent pregnancy to be associated with religious factors, early marriages, low level of education, and poverty. Also, cost of contraceptives, lack of adolescent-friendly health service provision, inadequate and unskilled health workers, and lack of comprehensive sexuality education.

Policymakers and opinion leaders should focus on community sensitization, comprehensive sexuality education and ensure girls enroll and stay in schools. Also, peers and significant others should be involved in designing interventional programs for adolescent pregnancy prevention. This could reduce adolescent pregnancy rates. Moreover, provision of adolescent-friendly health services at schools and healthcare centers, and initiating adolescent empowerment programs could have a positive impact on reducing adolescent pregnancy.

Further research is required on the determinants of adolescent pregnancy in the top five sub-Saharan African countries— Niger, Mali, Angola, Mozambique, and Guinea-- with rates of adolescent pregnancy above 140 births per 100,000 adolescent women.
